# Retraction: Role of metformin in suppressing 1,2-dimethylhydrazine-induced colon cancer in diabetic and non-diabetic mice: Effect on tumor angiogenesis and cell proliferation

**DOI:** 10.1371/journal.pone.0328096

**Published:** 2025-07-11

**Authors:** The PLOS One Editors

After publication of this article [1], concerns were raised about some of the results, methodology, and support for the conclusions. Specifically, in Fig 6A, the Combination 1 Diabetic panel appears to partially overlap with the Combination 2 Non-diabetic panel. In addition, an Editorial Board member raised concerns about the reliability of the quantitative results in Fig 6B given the methods used.

The corresponding author stated that the Combination 2 Non-diabetic panel is incorrect and provided a replacement panel for Combination 2 Non-diabetic stated to be from the slides from the time of the original experiments. The replacement panel did not appear well-matched to the other panels; it was reportedly imaged with different equipment. Overall, the Fig 6 issues are not fully resolved and PLOS remains concerned about the reliability of the Figure 6 results.

The *PLOS One* Editorial Board member also raised concerns that images shown in Fig 7A do not show exclusively nuclear staining as would be expected for Ki67 immunohistochemistry, and the counterstain is not visible in all panels. The Editorial Board member raised additional concerns about the methodology used for Ki67 experiments and advised that the data shown in Fig 7 do not support the quantitative claims made in the article about proliferation in treatment versus control groups.

In light of the above concerns and other issues noted in our editorial assessment, the *PLOS One* Editors retract this article due to concerns about the reliability of the reported results and conclusions.

SAZ did not agree with the retraction. DKZ and YMM either did not respond directly or could not be reached.

Note: The corresponding author noted an error in the units reported for serum VEGF and IGF-1 levels in [1]; the correct units for serum VEGF in Fig 3 and IGF-1 in Table 2 are ng/L.

## References

[1] Zaafar DK, Zaitone SA, Moustafa YM. Role of metformin in suppressing 1,2-dimethylhydrazine-induced colon cancer in diabetic and non-diabetic mice: effect on tumor angiogenesis and cell proliferation. PLoS One. 2014;9(6):e100562. doi: 10.1371/journal.pone.0100562 24971882 PMC4074064

